# An Analytical Compendium of the Varions Branches of Medical Science

**Published:** 1856-07

**Authors:** 


ART. IX. — *An Analytical Compendium of the Various Branches of Medical Science, for the use and examination of Students.* By JOHN NEILL, M. D., Surgeon to the Pennsylvania Hospital, Fellow of the College of Physicians, etc., and FRANCIS GURNEY SMITH, M. D., Physician to the St. Joseph's Hospital, Fellow of the College of Physicians, etc. A new edition, Revised and Improved. Philadelphia: BLANCHARD & LEA. 1856.

"Neill and Smith's Compend," is too well known to need any lengthy notice. Its merit is that of a condensed summary of the medical sciences, not full in any department, and not intended to take the place of text-books, but very useful to the student. The task of constructing such a work is a difficult one, but is here successfully carried out.

This kind of works as a class is objectionable, but the "Compend" of itself is a very meritorious book, and one which deserves the liberal patronage it has received. It can hardly be improved upon, except as successive editions may change the method of treating subjects which are advancing.

